# Maternal perinatal depression and child neurocognitive development: A relationship still to be clarified

**DOI:** 10.3389/fpsyt.2023.1151897

**Published:** 2023-03-20

**Authors:** Melania Severo, Antonio Ventriglio, Antonello Bellomo, Salvatore Iuso, Annamaria Petito

**Affiliations:** ^1^Department of Humanistic Studies, University of Foggia, Foggia, Italy; ^2^Department of Clinical and Experimental Medicine, University of Foggia, Foggia, Italy

**Keywords:** perinatal depression, neurocognitive development, post-partum depression, mother–child relations, maternal depression trajectories

## Abstract

Pregnancy frequently is associated with emotional conditions such as anxiety and depression. Perinatal depression has an incidence of around 12%. Only recently researcher put the attention on the effects of pre- and postpartum psychopathology on infant neurocognitive development. Neurobiology studies indicate that perinatal maternal depression can significantly affect the structure and function of children’s prefrontal cortex and modulate the development of cognitive abilities from intrauterine life. On the topic, the scientific literature appears ambiguous, reporting mixed results. Some studies have found no significant differences in developmental outcomes between prenatal and postpartum exposure to maternal depression, others have suggested a greater burden of depression in pregnancy than in postpartum, and still others have emphasized the role of chronicity of symptoms rather than the period of onset. Few studies have examined the effects of different developmental trajectories of maternal depression on children’s neurocognitive outcomes. The assessment of maternal health has for years been limited to postpartum depression often neglecting the timing of onset, the intensity of symptoms and their chronicity. These aspects have received less attention than they deserve, especially in relation to the effects on children’s neurocognitive development. The aim of this Perspective was to highlight inconsistencies and gaps that need to be filled in the approach to the study of this problem. Given the wide heterogeneity of data in the current literature, further studies are needed to clarify these interactions. This Perspective provides an overview of current progress, future directions, and a presentation of the authors’ views on the topic.

## 1. Introduction

Motherhood is a period of physical, psychological, and relational transformations that involve a deep reorganization not only of the external reality, but especially of the psychological world of the mother-to-be (1). Pregnancy is associated with changes in women’s psychological and relational functioning and can generate certain psychological conditions, such as feelings of anxiety and depression, even after childbirth (2, 3). At the biological level, pregnancy involves hormones (such as estrogen, progesterone, glucocorticoids, prolactin, and oxytocin) that can alter brain functioning and serve as a substrate to prepare women for the challenge of motherhood (4). After birth, there are not only new hormonal changes, but also other psychological and social factors such as alterations in intimate relationships, confrontation with the physical changes, breastfeeding, lack of social support, in a condition that is already emotionally vulnerable (5). A recent systematic review and meta-analysis showed that the prevalence of postpartum depression among healthy women who have never had depressive episodes stands at 17% (6). Postpartum depression is a mood disorder that usually has onset within the first 4–6 weeks after delivery and lasts up to a year, reaching its maximum intensity within the first 6 months (7). Surprisingly, a recent study by Putnick et al. (8) conducted in a sample of 4,866 women from the general population shows that depressive symptoms can be elevated up to 3 years after childbirth. Very often postpartum depression is a continuation of depressive symptoms already present in the prenatal period and is closely related to the presence of anxiety symptoms during pregnancy (9). More recent research is directing attention to perinatal depression, which considers both depressive manifestations arising during pregnancy and after delivery. In a systematic review and meta-regression of the prevalence of perinatal depression Woody et al. (10) indicate that the global prevalence of perinatal depression has been estimated at 11.9%, although significant differences emerge between high-and low-income countries (6). Perinatal depression is a significant mental and public health problem (11), not only because of its high frequency, but also because it is associated with adverse consequences for future maternal and child health (12). These consequences range from low birth weight or premature birth to the development of insecure attachment, problems in social–emotional, neurocognitive, language, and motor development (12–14). The perinatal period is characterized by special growth and sensitivity. Therefore, intrauterine exposures and early experiences may influence fetal and infant development with long-term consequences (12). While the influences of maternal perinatal psychopathology on children’s emotional and relationship development have been extensively investigated (15–18), fewer studies have focused on its effects on neurocognitive development, providing unclear results (19, 20). The purpose of this perspective was to identify weaknesses and aspects that need to be addressed in the approach to the study of these phenomena. Due to the heterogeneity of the data in the current literature, further studies are needed. This perspective gives an overview of current progress, gaps to be filled and future directions, and provides the authors’ views on the subject.

## 2. Search strategy

Even though it was not our goal to conduct a literature review, we tried to provide an overview including reviews, meta-analyses, and recent articles on the subject. We planned two steps in the literature search. First, relevant publications were found by searching the Pubmed electronic databases. The following search strategy was used for the Pubmed database: ((((Perinatal[Title/Abstract] OR postnatal [Title/Abstract] OR postpartum[Title/Abstract] OR antenatal[Title/Abstract] OR maternal[Title/Abstract]) AND (child*[Title/Abstract] OR infant*[Title/Abstract])) AND (neurocognit*[Title/Abstract] OR neurodevelopment*[Title/Abstract] OR development[Title/Abstract])) AND (depression[Title/Abstract])). We selected recent literature to identify current advances in the field of interest. Based on a qualitative assessment, the studies considered most useful in highlighting contradictions in the literature data were selected. In the second phase, the snowballing search method was used to track down related works using the bibliography or reference list at the end of the most interesting articles - especially reviews and meta-analyses - found in the first phase of the search.

## 3. Perinatal depression and child neurocognitive development

Neurocognitive development refers to the full range of mental activities and abilities, including memory, language, learning, problem solving, perception, and higher abilities such as executive functions involving attention and behavioral control, which are important for self-regulation (21). Specifically, executive functions refer to a set of cognitive and self-regulatory processes such as working memory, inhibitory control, and flexibility of attention necessary to plan future behaviors, make predictions, enact goal-oriented behaviors, and adapt to novelty (22, 23). In close correspondence with the rapid maturation of the brain and prefrontal cortex (24), neurocognitive development accelerates in the second half of the first year, leading to improvements in executive performance between 8 and 12 months. Importantly, these abilities are particularly flexible (15, 25) because the prefrontal cortex, which underlies the development of executive functions, shows plasticity and sensitivity to early care and experiences. In addition, maturation of the prefrontal cortex and other involved brain structures occurs over a long period of time, making cognitive functioning open to positive environmental input, but at the same time vulnerable to early adversity (20, 24). Alterations in executive function have been shown to cause lifelong effects, such as problem behavior in preschool (26), higher incidence of specific learning disorders (27), internalizing and externalizing disorders in childhood (28), school dropout (29), and lower academic achievement (30). Prenatal stress has been related to higher anxiety, depression, larger amygdala volume, and altered neural connectivity in girls (31–34) and to higher frequency of disturbances or delays in neuromuscular and cognitive development in general (34, 35). Assessments conducted on children’s nonverbal communication skills at age 14 months also indicate that maternal depressive symptomatology is associated with an increased risk of developmental delay (36). The cognitive development of 6-to 8-week-old infants (37) and the cognitive, language, and motor development of 4- and 13-month-olds (38) with depressed mothers is also lower when measured with the Bayley Scales of Infant and Toddler Development. Children of depressed mothers are six times more likely to be at risk for delayed emotional development (39) and five times more likely to be at risk for delayed language development with negative impact also on cognitive development at 6 months and 12 months and on motor development at 12 months and 18 months (39). Longitudinal studies indicate that maternal perinatal depression is associated with alterations in executive functions such as inhibition, shifting ability, cognitive flexibility, and working memory (12, 19, 20), but also with decreased thickness of the right frontal cortex (40, 41). In a study by Park et al. (42), the neurodevelopment outcomes of children of women with partially improved depressive symptoms in the first 3 years after delivery were comparable to those of children of mothers who consistently reported low levels of depressive symptomatology. In contrast, in women who continued to have more severe and longer-lasting depressive symptoms, children had greater neurocognitive impairments. This would suggest that an improvement in the mothers’ symptomatology in the first 3 years would reduce, or even reverse, the impact of maternal psychopathology on the children. Moreover, Faleschini et al. (43) found that exposure to prenatal maternal depressive symptoms predicts in children poorer executive functions related to planning, organization, working memory, inhibition of inappropriate impulses, emotional control, and ability to reevaluate and modify responses. At the same time, the authors, after accounting for influential factors including depressive symptoms in the prenatal period, identified poor associations between maternal depressive symptoms at six and 12 months postpartum and development in middle childhood. A recent meta-analysis (20) found no significant differences in neurocognitive developmental outcomes between prenatal and postpartum exposure to maternal depression, highlighting the need for further studies measuring depression over a longer time frame and emphasizing the importance of assessing chronicity of depressive symptomatology. Ibanez et al. (44) found that prenatal maternal depression is not associated with child development except when prenatal maternal anxiety is also present, while postpartum maternal depression would appear to play a mediating role in the relationship between prenatal maternal anxiety and children’s cognitive development. Evaluations conducted on the outcomes of prenatal, postpartum depression and their combination suggest that only prenatal depression is a significant predictor of cognitive development in children (45), thus the impact of prenatal depression cannot be explained by postpartum depressive symptoms. Indeed, in a very large study of 3,379 mother-infant pairs, prenatal maternal depression was associated with less optimal early neurodevelopment of the child, including social–emotional, cognitive, and motor skills in the first 2 years, which were not observed for postpartum depression (46). Also, in low-and middle-income countries has been shown up a direct association between prenatal depression and child cognitive development to the second and third years, even independent of the presence of postpartum depression (19). In a study by Urizar (47) maternal depression was assessed during pregnancy and 6 months after delivery, while infant development was assessed up to 5 years after delivery. The results showed that maternal depression experienced during pregnancy was associated with less cognitive development of the child. However, both the onset in pregnancy and early postpartum and the severity and chronicity of depression were associated with lower social–emotional development. Although few studies have examined the effects of severity and chronicity of maternal depressive symptoms beyond the perinatal period on child outcomes, research shows that depressive symptoms can persist long after birth (8), meaning many children in early childhood are exposed to maternal depression. As early as 2000, a study by Brennan et al. (48) suggested that the interaction between the severity and chronicity of maternal depressive symptoms are related to more behavioral problems and worse language performance in children, concluding that greater severity of depressive symptoms is likely to be accompanied by worse infant developmental outcomes. Interestingly, one study has explored and identified three developmental trajectories of maternal depressive symptoms from pregnancy to 4 years after delivery (49) describing them as follows: (1) no or few symptoms (61%), (2) persistent subclinical symptoms (30%) and (3) persistently elevated escalating symptoms (9%). Specifically, the authors found that children of mothers with subclinical, escalating, and persistently elevated symptoms were at least twice as likely to experience emotional and relationship difficulties than the others, even after accounting for other risk factors. Despite this, studies still seem to overlook the importance and interaction between severity, chronicity, and time of onset of maternal depression in predicting children’s outcomes. Similarly, there is little clarity on the role of childhood age of exposure to maternal depression. Overall, as argued in the discussion section, these results highlight the need to prospectively examine the developmental trajectories of maternal depression, giving greater relevance to the times of onset of depression (prenatal or postpartum), to the chronicity and severity of depressive symptoms and paying more attention to their effect on subsequent outcomes of the child’s neurocognitive development.

## 4. Mechanisms underlying the interaction between maternal perinatal depression and child neurocognitive development

There are multiple mechanisms that might underlie the interactions mentioned above. First, mothers with depressive symptoms might initiate with greater difficulty a dyadic interaction within which to maintain joint attention with their child. Consistent with other authors, we consider it is possible that these women are more distracted and less attuned to their children’s demands for interaction (50). Indeed, postpartum depression has been associated with maternal disengagement (12). Mothers who exhibit depressive symptoms perform less verbal repetition, give fewer explanations and suggestions, and thus have reduced lexical input (50). They also appear more withdrawn and less attuned, showing a narrow range of emotional expressions and being emotionally unpredictable (15, 51). Although the biological aspects underlying these influences are not yet entirely identified, they could involve changes in hormone regulation and gene expression in the placenta (52). Among other mechanisms, studies suggest that prenatal depression might lead to epigenetic dysregulation in offspring through alterations in the hypothalamic–pituitary–adrenal axis and serotonergic transmission (12). Neurobiology studies indicate that prenatal maternal stress can significantly affect the structure and function of the prefrontal cortex due to the rapid brain development during pregnancy and due to the high density of glucocorticoid receptors in the prefrontal cortex (40, 53). Alterations in white matter of 2-3-year-old children with prenatal exposure to depression could explain functional impact on cognitive development (54), moreover other structural and microstructural changes have also been described in the neonatal period (55). Scientific evidence suggests that exposure to maternal depression, both prenatal and postpartum, influences children’s lifelong health and illness pathways. Continued exposure to maternal depression in early life seems an independent risk factor for poorer cognitive, behavioral, and emotional development during childhood and adolescence (12). Notably, these effects appear to persist even when controlling for other risk variables such as obstetric complications or psychosocial conditions (42).

## 5. The role of psychological and psychosocial factors

Recently, maternal stress and anxiety have been recognized as important as depressive states. The prevalence of anxiety disorders during pregnancy is around 10% in developed countries and about 25% in developing countries (56). Buss et al. (57) report that pregnancy-specific anxiety is associated with lower visual–spatial working memory in both sexes and lower inhibitory control in girls. A recent review by Fitzgerald et al. (58) suggests that changes in DNA methylation are present in the umbilical cord blood and in the blood of children of mothers affected by prenatal stress. Similarly, inflammatory mechanisms and processes are also powerful modifiers of neurodevelopment and infant behavior. Other factors are also important and should be considered. For instance, Gonzalez et al. (59) found that economic, psychosocial, physiological and perinatal well-being were associated with better cognitive performance and greater total cortical surface area in children. Regarding socioeconomic status, lower maternal socioeconomic status has been found to negatively influence cognitive and behavioral outcomes at age 24 months (60) as well as the brain structure of children and their language performance. Few studies have examined these issues in low-and middle-income settings, where children are generally exposed to a greater number and range of adversities. A systematic review by Fisher et al. (61) highlighted socioeconomic disadvantage, unwanted pregnancy, young age, being unmarried, lack of empathy and support from partner, the presence of hostile in-laws, experience of violence by an intimate partner, insufficient emotional and practical support, and a history of mental health problems are among the most important risk factors for perinatal mental disorders in women from low-and middle-income countries. The authors concluded that perinatal mental disorders are more prevalent in low-and middle-income countries, particularly among poorer women with gender risk or psychiatric history. According to Bluett-Duncan et al. (19), the co-presence of additional risk factors in low-and middle-income settings may result in more impaired development than observed in middle-and upper-income socio-cultural settings. This suggests that socioeconomic adversity within low-income countries plays a significant role in determining the burden of perinatal depression on child development, making opportunities to recover from any developmental alterations less frequent. More studies are needed in low-to middle-development areas. Among other factors, pre and postnatal exposure to tobacco may also influence children neurocognitive development. For example, children of mothers who smoked during pregnancy may show delays in motor function and may exhibit less cognitive abilities in attention and response inhibition (62). Moreover, child smoking exposure *in utero* is associated with lower total brain volume and brain gray matter volume in preadolescence (63). Furthermore, maternal exposure to drugs revealed pronounced effects on executive functions and mild impairment of spatial memory in children (64). Alcohol, nicotine, cocaine, amphetamine, ecstasy, and opioids have been shown to produce alterations in neurodevelopmental trajectories (65). A recent systematic review has shown that prenatal alcohol exposure impacts motor skills, cognition, language, school achievement, attention, and affect regulation (66). However, further studies examining substances consumption and subsequent neurodevelopmental outcomes are needed (67). We could argue that health services should consider implementing maternal-perinatal health screening programs, collecting more timely and accurate information on sometimes overlooked risk factors. In addition, more in-depth assessment programs would be important for children most exposed *in utero* to the above risk factors.

## 6. Discussion

According to other authors (19, 42), we consider that the existing literature is insufficient to distinguish the effect of prenatal versus postpartum maternal depressive symptoms on children’s development. It is possible that it is the intensity and chronicity of maternal depression, rather than the timing of exposure (pre or postpartum) that plays a critical role in influencing children’s mental health and development. Although several studies indicate an association between pre-and postnatal depression and infant neurodevelopmental outcomes, there is currently limited agreement on the period most susceptible to this influence. Several studies have shown that maternal depression during pregnancy, but not after birth, predicts adverse effects in child (45, 46, 68). Exposure to prenatal maternal depressive symptoms predicts in children poorer executive functions related to planning, organization, working memory, inhibition of inappropriate impulses and emotional control (43). However, poor associations were identified between maternal depressive symptoms at six and 12 months postpartum and neurodevelopment in middle childhood (43). Evaluations conducted on the outcomes of prenatal, postpartum depression and their combination suggest that only prenatal depression is a significant predictor of cognitive development in children (19, 20, 45). Therefore, the evidence for the direct influence of postpartum depression on cognitive development is more equivocal (19, 43, 44). To explain this inconsistency in results, we could argue how difficult it is to quantify and circumscribe the onset and duration of depressive symptoms using self-report instruments, usually administered at one time. The Edinburg Postnatal Depression Scale by Cox et al. (69), a validated and reliable instrument, is widely used in maternal depression research, but still a self-report scale that does not allow for diagnosis. EPDS also neglects intensity and stability of symptoms over time. As a result, most studies rely on the level of depressive symptoms and not on diagnoses of depressive disorders to assess the impact of maternal depression on child development. Moreover, in past decades, maternal mental health screenings were mostly done in the postpartum period, not discriminating the period of onset of the disorder. In addition, women are often unable to precisely detect the period of occurrence because they are long-standing disorders. Assessments of maternal mood based only on one or two assessments in the first year after delivery or on retrospective assessments thereafter are subject to recall bias. This type of assessment also makes it difficult to distinguish the effect of depressive symptoms developed in the first few weeks after birth from those developed several months after the child’s birth. The method in which the child’s development is assessed, either with standardized instruments or with parent-reported assessments, could also influence the results of a study. Furthermore, many studies tend to assess children’s neurocognitive development at only one follow-up, resulting in a serious loss of information. There is considerable heterogeneity in the measurement of child neurocognitive development. The most widely used instruments for neurocognitive assessment include the Bayley Scales of Infant and Toddler Development-Third Edition (BSID-III; 70), the Wechsler Intelligence Scale for Children (WISC, 71), various cognitive tasks (e.g., sequential memory tests, working memory tasks, inhibition tasks, etc.), heterogeneous neuropsychological batteries to assess executive functions or data collected from parents (e.g., Behavior Rating Inventory of Executive Function - Preschool version, BRIEF-P, 72). The BSID-III (70) is the most widely used standardized assessment of cognitive, language and motor development in infants aged 6 to 42 months. This instrument has good cross-cultural validity and has greater sensitivity to changes in development. However, it is an expensive and time-consuming instrument to administer. Several studies have used parent self-report assessments of child development. Although self-report measures have the advantage of providing information based on the natural life context at a reduced cost, the exclusive use of self-report measures could lead to bias in conclusions about child development (19). Indeed, maternal self-report measures are likely to be biased by mood, making ratings more negative due to negative cognitive biases common in depression. Given that the heterogeneity of data in the literature suggests that measured effects may be sensitive to instrument choice, an important step will be the selection or development of feasible and culturally sensitive measures of cognitive development (19). Future studies would benefit from the use of both physician-administered diagnostic interviews and developmental assessment and observation batteries, which, on the other hand, are more time-consuming. In addition, most of the research to date has focused primarily on early child development, single assessments or very limited time follow-ups. Future research could explore a longer time frame to strengthen the evidence about different developmental stages (12). However, mothers with higher depressive symptoms may be less likely to participate in longer follow-ups over time, with increased risk of drop out. Many studies base their assessments solely on childhood executive functions. Several authors have argued that executive functions might reflect an ability distinct from other measures of neuropsychological development (73). Therefore, it can be hypothesized that the effects of maternal depression on executive functions might be independent of overall cognitive development. This observation underscores the need to use of instruments that investigate the relationship of maternal depression with overall cognitive development and not just executive functions. The absence of clinical information on the characteristics and history of depressive symptoms and the relative paucity of longitudinal studies on children of depressed mothers make it difficult to characterize the nature of depression and its outcomes. In our opinion, although some studies have investigated the relationship between maternal depression in the postpartum period and children’s neurocognitive development, few studies have really focused on the effect of different trajectories of maternal depression from the prenatal period to the postpartum period. Similarly, research differs in terms of the timing of assessments in the gestational period or postpartum period, and consequently it is difficult to compare them. In addition, the sample sizes of longitudinal studies on cohorts of mothers and children are probably insufficient to represent the magnitude of the phenomenon in the population examined. Further research is needed to clarify these issues. Depression in women is a serious public health problem, and in past years the focus has been mainly on depression in the postpartum period. Putting light on the trajectories of influence of prenatal and postpartum depression both combined and independently could be of crucial importance in identifying mother–child dyads at higher developmental risk and in developing early intervention programs from the earliest stages of pregnancy. If prenatal depression persists after childbirth, it may affect the quality of parental functions and parental care, as well as the woman’s perception of the newborn itself. Thus, an inconsistent style of care might undermine the relational foundations that should promote the maturation of self-regulation and higher-order cognitive skills in child. These effects may be even more pronounced among the offspring of chronically depressed mothers. It seems clear that screening for perinatal depression should be an important goal to support maternal and child mental health. Unfortunately, perinatal depression is often undiagnosed or untreated. The results from a systematic literature review on detection and treatment rates of perinatal depression indicate that approximately 50% of women with prenatal depression are identified in the clinical setting, of these 14% receive treatment and only 8.6% receive adequate treatment (58). For women with postpartum depression, about 31% are identified, 16% receive treatment and only 6.3% receive adequate treatment (74). Clinically, these data suggest that it would be wrong to focus psychological interventions exclusively on depressed mothers in the early postpartum weeks. Early intervention during pregnancy could have important clinical implications reducing the impact of psychopathology on maternal and child health. Even if the possible efficacy of different interventions has yet to be explored, a very recent study indicates that cognitive-behavioral psychotherapy interventions on depression during pregnancy were shown to be effective in indirectly improving infant motor development during the first 18 months after birth (75). Understanding the prenatal and postpartum mechanisms underlying these relationships would help design more appropriate timing of successful prenatal and/or postpartum interventions, as well as identifying a gestational age of increased vulnerability would change the direction of research in clinical practice. The Authors of this perspective consider that it is not the presence of depressive symptoms *per se* at a given stage of pregnancy or postpartum that influences the outcome of child neurodevelopment. We argue that it is the pervasiveness and intensity of this symptomatology over a long period of time that determines the negative effects on neurodevelopment. We also believe that future investigations should consider a broader time window capable of discerning the exerted influences of pre-and postpartum depression, using not only self-report instruments but also clinical interviews. In addition, future research should more accurately consider the influence of psychosocial risk factors, such as maternal stress, perceived social support, socioeconomic status, parental educational level, presence of a supportive partner, substance use and abuse (from tobacco to drugs) or the presence of stressful events in the past year on the child’s neurocognitive development, not just using them as control variables. In addition, more data should be collected on low and middle developing countries. Further studies should also consider other confounding factors, e.g., maternal IQ, being primipara, gender of the child, sociocultural background of the woman, and the presence of additional ongoing psychological symptomatology. In our idea, chronicity and intensity of maternal depression have greater negative effects on child neurodevelopment than a single depressive episode pre-and/or postpartum. Although a literature review was not within our objectives for this perspective, we tried to include data from a wide range of studies (including literature reviews and recent and original articles on the topic). This allowed us to outline the state of the art on the topic covered in this perspective, as well as to highlight inconsistencies and gaps that need to be filled in the approach to the study of this issue. Systematic reviews and meta-analyses on the topic have shown unclear results, therefore, a continuous evolution of research is needed in order to carry out a more careful analysis of the overall results in the literature. In this perspective, we pointed out that the assessment of maternal health has for years been limited to postpartum depression (and mostly to its influences on infant emotional and affective development), often neglecting the timing of its onset, the intensity of symptoms and their chronicity. In our idea, these aspects have received less attention than they deserve, considering the decisive role these factors might play in delineating the impact of maternal depression on childhood neurocognitive development. Certainly, as mentioned above, some aspects are very difficult to assess, thus they are less considered in the literature. However, recent studies have opened this discussion by highlighting the gaps and inconsistencies among the various data available in the literature. These considerations should be integrated in the evaluation and development of screening and treatment activities for the pregnant population, especially considering the actual burden in terms of health and disease development pathways.

## 7. Conclusion

Maternal depression represents a public health problem related to serious difficulties for the establishment of a good mother–child relationship. Maternal depression is also capable of directly and indirectly altering infant brain and neurocognitive development. Few prospective studies have examined the effects of perinatal depression on cognitive development with seemingly contradictory results. These inconsistencies may reflect methodological differences, but also the neglect of aspects such as the time of onset of depressive symptoms, their intensity and their duration. Further studies are needed to clarify these interactions. Understanding the prenatal and postpartum mechanisms underlying these relationships, as well as identifying a gestational age of increased vulnerability would help design more appropriate interventions. There is a clear need for clinicians to design early intervention pathways to be implemented already during pregnancy. These could have important clinical implications for mother–child dyads who are candidates for individualized psychological treatment aimed at reducing the impact of maternal psychopathology on child development.

## Data availability statement

The original contributions presented in the study are included in the article/supplementary material, further inquiries can be directed to the corresponding author.

## Author contributions

MS developed the theoretical formalism. All authors contributed to the article and approved the submitted version.

## Funding

This project has been supported by the Department of Health Promotion of the Regione Puglia, Italy.

## Conflict of interest

The authors declare that the research was conducted in the absence of any commercial or financial relationships that could be construed as a potential conflict of interest.

## Publisher’s note

All claims expressed in this article are solely those of the authors and do not necessarily represent those of their affiliated organizations, or those of the publisher, the editors and the reviewers. Any product that may be evaluated in this article, or claim that may be made by its manufacturer, is not guaranteed or endorsed by the publisher.

## References

[1] Johnston-Ataata K Kokanovic R Michaels PA. One of the most vulnerable times in your life: expectations and emotional experiences of support in the early postnatal period In: Kokanovic R Michaels P Johnston-Ataata K, editors. Paths to parenthood: Emotions on the journey through pregnancy, childbirth, and early parenthood. 1st ed. London: Palgrave Macmillan (2018)

[2] Meems M Hulsbosch L Riem M Meyers C Pronk T. Broeren M, he Brabant study: design of a large prospective perinatal cohort study among pregnant women investigating obstetric outcome from a biopsychosocial perspective. BMJ Open. (2020) 10:e038891. doi: 10.1136/bmjopen-2020-038891, PMID: 33109659 PMC7592269

[3] van de Loo KFE Vlenterie R Nikkels SJ Merkus PJFM Roukema J Verhaak CM. Depression and anxiety during pregnancy: the influence of maternal characteristics. Birth. (2018) 45:478–9. doi: 10.1111/birt.12343, PMID: 29517137

[4] Stolzenberg DS Champagne FA. Hormonal and non-hormonal bases of maternal behavior: the role of experience and epigenetic mechanisms. Horm Behav. (2016) 77:204–08. doi: 10.1016/j.yhbeh.2015.07.005, PMID: 26172856

[5] Lagadec N Steinecker M Kapassi A Magnier AM Chastang J Robert S. Factors influencing the quality of life of pregnant women: a systematic review. BMC Pregnancy Childbirth. (2018) 18:455. doi: 10.1186/s12884-018-2087-4, PMID: 30470200 PMC6251086

[6] Shorey S Chee CYI Ng ED Chan YH Tam WWS Chong YS. Prevalence and incidence of postpartum depression among healthy mothers: a systematic review and meta-analysis. J Psychiatr Res. (2018) 104:235–8. doi: 10.1016/j.jpsychires.2018.08.001, PMID: 30114665

[7] Silva CS Lima MC Sequeira-de-Andrade LAS Oliveira JS Monteiro JS Lima NMS. Association between postpartum depression and the practice of exclusive breastfeeding in the first three months of life. J Pediatr. (2017) 93:356–64. doi: 10.1016/j.jped.2016.08.005 28034730

[8] Putnick DL Sundaram R Bell EM Ghassabian A Goldstein RB Robinson SL. Trajectories of maternal postpartum depressive symptoms. Pediatrics. (2020) 146:e20200857. doi: 10.1542/peds.2020-0857, PMID: 33109744 PMC7772818

[9] Pearson RM Bornstein MH Cordero M Scerif G Mahedy L Evans J. Maternal perinatal mental health and offspring academic achievement at age 16: the mediating role of childhood executive function. J Child Psychol Psychiatry. (2016) 57:491–1. doi: 10.1111/jcpp.12483, PMID: 26616637 PMC4789117

[10] Woody CA Ferrari AJ Siskind DJ Whiteford HA Harris MG. A systematic review and meta-regression of the prevalence and incidence of perinatal depression. J Affect Disord. (2017) 219:86–92. doi: 10.1016/j.jad.2017.05.003, PMID: 28531848

[11] Shrivastava SR Shrivastava PS Ramasamy J. Antenatal and postnatal depression: a public health perspective. J. Neurosci. Rural Pract. (2015) 6:116–9. doi: 10.4103/0976-3147.143218, PMID: 25552868 PMC4244771

[12] Rogers A Obst S Teague SJ Rossen L Spry EA Macdonald JA. Association between maternal perinatal depression and anxiety and child and adolescent development: a meta-analysis. JAMA Pediatr. (2020) 174:1082–92. doi: 10.1001/jamapediatrics.2020.2910, PMID: 32926075 PMC7490743

[13] Rosenthal L Earnshaw VA Moore JM Ferguson DN Lewis TT Reid AE. Intergenerational consequences: Women’s experiences of discrimination in pregnancy predict infant social-emotional development at 6 months and 1 year. J Dev Behav Pediatr. (2018) 39:228–7. doi: 10.1097/DBP.0000000000000529, PMID: 29176360 PMC5866165

[14] Hayes LJ Goodman SH Carlson E. Maternal antenatal depression and infant disorganized attachment at 12 months. Attach Hum Dev. (2013) 15:133–3. doi: 10.1080/14616734.2013.743256, PMID: 23216358 PMC3594350

[15] Priel A Djalovski A Zagoory-Sharon O Feldman R. Maternal depression impacts child psychopathology across the first decade of life: oxytocin and synchrony as markers of resilience. J Child Psychol Psychiatry. (2019) 60:30–42. doi: 10.1111/jcpp.12880, PMID: 29484656

[16] Esteves KC Jones CW Wade M Callerame K Smith AK Theall KP. Adverse childhood experiences: implications for offspring telomere length and psychopathology. Am J Psychiatry. (2020) 177:47–57. doi: 10.1176/appi.ajp.2019.18030335, PMID: 31509004 PMC7273739

[17] Gjerde LC Eilertsen EM Hannigan LJ Eley T Røysamb E Reichborn-Kjennerud T. Associations between maternal depressive symptoms and risk for offspring early-life psychopathology: the role of genetic and non-genetic mechanisms. Psychol Med. (2021) 51:441–9. doi: 10.1017/S0033291719003301, PMID: 31813389

[18] Hentges RF Graham SA Plamondon A Tough S Madigan S. Bidirectional associations between maternal depression, hostile parenting, and early child emotional problems: findings from the all our families cohort. J Affect Disord. (2021) 287:397–4. doi: 10.1016/j.jad.2021.03.056, PMID: 33838474

[19] Bluett-Duncan M Kishore MT Patil DM Satyanarayana VA Sharp H. A systematic review of the association between perinatal depression and cognitive development in infancy in low and middle-income countries. PLoS One. (2021) 16:e0253790. doi: 10.1371/journal.pone.0253790, PMID: 34170948 PMC8232443

[20] Power J van Ijzendoorn M Lewis AJ Chen W Galbally M. Maternal perinatal depression and child executive function: a systematic review and meta-analysis. J Affect Disord. (2021) 291:218–4. doi: 10.1016/j.jad.2021.05.003, PMID: 34049191

[21] Kihlstrom JF. Unconscious Cognition. Reference module in neuroscience and biobehavioral psychology. Amsterdam: Elsevier (2018).

[22] Snyder HR Miyake A Hankin BL. Advancing understanding of executive function impairments and psychopathology: bridging the gap between clinical and cognitive approaches. Front Psychol. (2015) 6:328. doi: 10.3389/fpsyg.2015.00328, PMID: 25859234 PMC4374537

[23] Nolvi S Pesonen H Bridgett DJ Korja R Kataja EL Karlsson H. Infant sex moderates the effects of maternal pre-and postnatal stress on executive functioning at 8 months of age. Infancy. (2017) 23:194–08. doi: 10.1111/infa.12206

[24] Hodel AS. Rapid infant prefrontal cortex development and sensitivity to early environmental experience. Dev Rev. (2018) 48:113–4. doi: 10.1016/j.dr.2018.02.003, PMID: 30270962 PMC6157748

[25] Zelazo PD Carlson SM. Hot and cool executive function in childhood and adolescence: development and plasticity. Child Dev Perspect. (2012) 6:354–08. doi: 10.1111/j.1750-8606.2012.00246.x

[26] Monette S Bigras M Guay MC. Executive functions in kindergarteners with high levels 299 of disruptive behaviours. Br J Dev Psychol. (2015) 33:446–3. doi: 10.1111/bjdp.12105, PMID: 26198079

[27] Operto FF Smirni D Scuoppo C Padovano C Vivenzio V Quatrosi G. Neuropsychological profile, emotional/behavioral problems, and parental stress in children with neurodevelopmental disorders. Brain Sci. (2021) 11:584. doi: 10.3390/brainsci11050584, PMID: 33946388 PMC8146823

[28] Hughes C Ensor R. Individual differences in growth in executive function across the transition to school predict externalizing and internalizing behaviors and self-perceived academic success at 6 years of age. J Exp Child Psychol. (2011) 108:663–6. doi: 10.1016/j.jecp.2010.06.005, PMID: 20673580

[29] Bierman KL Nix RL Greenberg MT Blair C Domitrovich CE. Executive functions and school readiness intervention: impact, moderation, and mediation in the Head start REDI program. Dev Psychopathol. (2008) 20:821–3. doi: 10.1017/S0954579408000394, PMID: 18606033 PMC3205459

[30] Bull R Espy KA Wiebe SA. Short-term memory, working memory, and executive functioning in preschoolers: longitudinal predictors of mathematical achievement at age 7 years. Dev Neuropsychol. (2008) 33:205–8. doi: 10.1080/87565640801982312, PMID: 18473197 PMC2729141

[31] Buss C Davis EP Shahbaba B Pruessner JC Head K Sandman CA. Maternal cortisol over the course of pregnancy and subsequent child amygdala and hippocampus volumes and affective problems. Proc Natl Acad Sci U S A. (2012) 109:E1312–9. doi: 10.1073/pnas.1201295109, PMID: 22529357 PMC3356611

[32] Kim DJ Davis EP Sandman CA Sporns O O’Donnell BF Buss C. Prenatal maternal cortisol has sex-specific associations with child Brain network properties. Cereb Cortex. (2017) 27:5230–41. doi: 10.1093/cercor/bhw303, PMID: 27664961 PMC6084613

[33] Quarini C Pearson RM Stein A Ramchandani PG Lewis G Evans J. Are female children more vulnerable to the long-term effects of maternal depression during pregnancy? J Affect Disord. (2016) 189:329–5. doi: 10.1016/j.jad.2015.09.039, PMID: 26469300 PMC4650986

[34] Sandman CA Glynn LM Davis EP. Is there a viability-vulnerability tradeoff? Sex differences in fetal programming. J Psychosom Res. (2013) 75:327–5. doi: 10.1016/j.jpsychores.2013.07.009, PMID: 24119938 PMC3796732

[35] Ellman LM Schetter CD Hobel CJ Chicz-Demet A Glynn LM Sandman CA. Timing of fetal exposure to stress hormones: effects on newborn physical and neuromuscular maturation. Dev Psychobiol. (2008) 50:232–1. doi: 10.1002/dev.20293, PMID: 18335490 PMC2851937

[36] Kawai E Takagai S Takei N Itoh H Kanayama N Tsuchiya KJ. Maternal postpartum depressive symptoms predict delay in non-verbal communication in 14-month-old infants. Infant Behav Dev. (2017) 46:33–45. doi: 10.1016/j.infbeh.2016.11.006, PMID: 27870989

[37] Liu Y Kaaya S Chai J McCoy DC Surkan PJ Black MM. Maternal depressive symptoms and early childhood cognitive development: a meta-analysis. Psychol Med. (2017) 47:680–9. doi: 10.1017/S003329171600283X, PMID: 27834159

[38] Smith-Nielsen J Tharner A Krogh MT Vaever MS. Effects of maternal postpartum depression in a well-resourced sample: early concurrent and long-term effects on infant cognitive, language, and motor development. Scand J Psychol. (2016) 57:571–3. doi: 10.1111/sjop.12321, PMID: 27611177

[39] Ali NS Mahmud S Khan A Ali BS. Impact of postpartum anxiety and depression on child's mental development from two peri-urban communities of Karachi Pakistan: a quasi-experimental study. BMC Psych. (2013) 13:274. doi: 10.1186/1471-244X-13-274, PMID: 24148567 PMC3819469

[40] Arnsten AF. Stress signalling pathways that impair prefrontal cortex structure and function. Nat Rev Neurosci. (2009) 10:410–2. doi: 10.1038/nrn2648, PMID: 19455173 PMC2907136

[41] Sandman CA Buss C Head K Davis EP. Fetal exposure to maternal depressive symptoms is associated with cortical thickness in late childhood. Biol Psychiatry. (2015) 77:324–4. doi: 10.1016/j.biopsych.2014.06.025, PMID: 25129235 PMC4289467

[42] Park M Brain U Grunau RE Diamond A Oberlander TF. Maternal depression trajectories from pregnancy to 3 years postpartum are associated with children's behavior and executive functions at 3 and 6 years. Arch Womens Ment Health. (2018) 21:353–3. doi: 10.1007/s00737-017-0803-0, PMID: 29340801

[43] Faleschini S Rifas-Shiman SL Tiemeier H Oken E Hivert MF. Associations of prenatal and postnatal maternal depressive symptoms with offspring cognition and behavior in mid-childhood: a prospective cohort study. Int J Environ Res Public Health. (2019) 16:1007. doi: 10.3390/ijerph16061007, PMID: 30897718 PMC6466510

[44] Ibanez G Bernard JY Rondet C Peyre H Forhan A Kaminski M. Effects of antenatal maternal depression and anxiety on Children's early cognitive development: a prospective cohort study. PLoS One. (2015) 10:e0135849. doi: 10.1371/journal.pone.0135849, PMID: 26317609 PMC4552796

[45] Tran TD Biggs BA Tran T Simpson JA Hanieh S Dwyer T. Impact on infants' cognitive development of antenatal exposure to iron deficiency disorder and common mental disorders. PLoS One. (2013) 8:e74876. doi: 10.1371/journal.pone.0074876, PMID: 24086390 PMC3781140

[46] Zhang T Luo ZC Ji Y Chen Y Ma R Fan P. The impact of maternal depression, anxiety, and stress on early neurodevelopment in boys and girls. J Affect Disord. (2023) 321:74–82. doi: 10.1016/j.jad.2022.10.030, PMID: 36280196

[47] Urizar GG Jr Muñoz RF. Role of maternal depression on child development: a prospective analysis from pregnancy to early childhood. Child Psychiatry Hum Dev. (2022) 53:502–4. doi: 10.1007/s10578-021-01138-1, PMID: 33646485 PMC10911822

[48] Brennan PA Hammen C Andersen MJ Bor W Najman JM Williams GM. Chronicity, severity, and timing of maternal depressive symptoms: relationships with child outcomes at age 5. Dev Psychol. (2000) 36:759–6. doi: 10.1037//0012-1649.36.6.759 11081699

[49] Giallo R Woolhouse H Gartland D Hiscock H Brown S. The emotional-behavioural functioning of children exposed to maternal depressive symptoms across pregnancy and early childhood: a prospective Australian pregnancy cohort study. Eur Child Adolesc Psychiatry. (2015) 24:1233–44. doi: 10.1007/s00787-014-0672-2, PMID: 25572869

[50] Gueron-Sela N Camerota M Willoughby MT Vernon-Feagans L Cox MJ. Family life project key investigators. Maternal depressive symptoms, mother-child interactions, and children’s executive function. Dev Psychol. (2018) 54:71–82. doi: 10.1037/dev0000389, PMID: 28933882 PMC5750080

[51] Granat A Gadassi R Gilboa-Schechtman E Feldman R. Maternal depression and anxiety, social synchrony, and infant regulation of negative and positive emotions. Emotion. (2017) 17:11–27. doi: 10.1037/emo0000204, PMID: 27441576

[52] Litzky JF Deyssenroth MA Everson TM Lester BM Lambertini L Chen J. Prenatal exposure to maternal depression and anxiety on imprinted gene expression in placenta and infant neurodevelopment and growth. Pediatr Res. (2018) 83:1075–83. doi: 10.1038/pr.2018.27, PMID: 29538358 PMC5959758

[53] Sanchez MM Young LJ Plotsky PM Insel TR. Distribution of corticosteroid receptors in the rhesus brain. J Neurosci. (2000) 20:4657–68. doi: 10.1523/JNEUROSCI.20-12-04657.2000, PMID: 10844035 PMC6772467

[54] Roos A Wedderburn CJ Fouche JP Joshi SH Narr KL Woods RP. Prenatal depression exposure alters white matter integrity and neurodevelopment in early childhood. Brain Imaging Behav. (2022) 16:1324–36. doi: 10.1007/s11682-021-00616-3, PMID: 35000066 PMC9107412

[55] Lautarescu A Bonthrone AF Pietsch M Batalle D Cordero-Grande L Tournier JD. Maternal depressive symptoms, neonatal white matter, and toddler social-emotional development. Transl Psychiatry. (2022) 12:323. doi: 10.1038/s41398-022-02073-y, PMID: 35945202 PMC9363426

[56] Shahhosseini Z Pourasghar M Khalilian A Salehi F. A review of the effects of anxiety during pregnancy on children’s health. Mater Socio Med. (2015) 27:200–2. doi: 10.5455/msm.2015.27.200-202, PMID: 26236168 PMC4499279

[57] Buss C Davis EP Hobel CJ Sandman CA. Maternal pregnancy-specific anxiety is associated with child executive function at 6–9 years age. Stress. (2011) 14:665–6. doi: 10.3109/10253890.2011.623250, PMID: 21995526 PMC3222921

[58] Fitzgerald E Parent C Kee M Meaney MJ. Maternal distress and offspring neurodevelopment: challenges and opportunities for pre-clinical research models. Front Neurosci. (2021) 15:635304. doi: 10.3389/fnhum.2021.635304, PMID: 33643013 PMC7907173

[59] Gonzalez MR Palmer CE Uban KA Jernigan TL Thompson WK Sowell ER. Positive economic, psychosocial, and physiological ecologies predict Brain structure and cognitive performance in 9-10-year-old children. Front Hum Neurosci. (2020) 14:578822. doi: 10.3389/fnhum.2020.578822, PMID: 33192411 PMC7655980

[60] Spann MN Bansal R Hao X Rosen TS Peterson BS. Prenatal socioeconomic status and social support are associated with neonatal brain morphology, toddler language and psychiatric symptoms. Child Neuropsychol. (2020) 26:170–8. doi: 10.1080/09297049.2019.1648641, PMID: 31385559 PMC6930975

[61] Fisher J. Cabral de Mello M, Patel V, Rahman a, Tran T, Holton S, et al. prevalence and determinants of common perinatal mental disorders in women in low-and lower-middle-income countries: a systematic review. Bull World Health Organ. (2012) 90:139G–49G. doi: 10.2471/BLT.11.091850, PMID: 22423165 PMC3302553

[62] Moore BF Shapiro AL Wilkening G Magzamen S Starling AP Allshouse WB. Prenatal exposure to tobacco and offspring neurocognitive development in the healthy start study. J Pediatr. (2020) 218:28–34.e2. doi: 10.1016/j.jpeds.2019.10.056 31759580 PMC7042047

[63] Zou R Boer OD Felix JF Muetzel RL Franken IHA Cecil CAM. Association of Maternal Tobacco use during Pregnancy with preadolescent Brain morphology among offspring. JAMA Netw Open. (2022) 5:e2224701. doi: 10.1001/jamanetworkopen.2022.24701, PMID: 35913739 PMC9344360

[64] Piper BJ Acevedo SF Kolchugina GK Butler RW Corbett SM Honeycutt EB. Abnormalities in parentally rated executive function in methamphetamine/polysubstance exposed children. Pharmacol Biochem Behav. (2011) 98:432–9. doi: 10.1016/j.pbb.2011.02.013, PMID: 21334365 PMC3069661

[65] Ross EJ Graham DL Money KM Stanwood GD. Developmental consequences of fetal exposure to drugs: what we know and what we still must learn. Neuropsychopharmacology. (2015) 40:61–87. doi: 10.1038/npp.2014.147, PMID: 24938210 PMC4262892

[66] Chu JTW McCormack J Marsh S Wells A Wilson H Bullen C. Impact of prenatal alcohol exposure on neurodevelopmental outcomes: a systematic review. Health Psychol Behav Med. (2022) 10:973–02. doi: 10.1080/21642850.2022.2129653, PMID: 36238426 PMC9553152

[67] Maher GM Khashan AS O'Byrne L Flanagan S Mortimer RM Kiely M. Periconceptual and prenatal alcohol consumption and neurodevelopment at age two and five years. Eur J Obstet Gynecol Reprod Biol. (2022) 274:197–3. doi: 10.1016/j.ejogrb.2022.05.034, PMID: 35667175

[68] Huot RL Brennan PA Stowe ZN Plotsky PM Walker EF. Negative affect in offspring of depressed mothers is predicted by infant cortisol levels at 6 months and maternal depression during pregnancy, but not postpartum. Ann N Y Acad Sci. (2004) 1032:234–6. doi: 10.1196/annals.1314.028, PMID: 15677418

[69] Cox JL Holden JM Sagovsky R. Detection of postnatal depression. Development of the 10-item Edinburgh postnatal depression scale. Br J Psychiatry. (1987) 150:782–6. doi: 10.1192/bjp.150.6.782 3651732

[70] Bayley N. Bayley scales of infant and toddler development-third edition: Administration manual. Harcourt Assessment: San Antonio, TX (2006).

[71] Wechsler D. Wechsler intelligence scale for children-fourth edition. San Antonio, TX: The Psychological Corporation (2003).

[72] Gioia GA Espy KA Isquith PK. Behavior rating inventory of executive function preschool version (BRIEF-P): Professional manual. Psychological Assessment Resources: Lutz, FL (2003).

[73] Sun J Mohay H O’Callaghan M. A comparison of executive function in very preterm and term infants at 8 months corrected age. Early Hum Dev. (2009) 85:225–08. doi: 10.1016/j.earlhumdev.2008.10.00572, PMID: 19006652

[74] Cox EQ Sowa NA Meltzer-Brody SE Gaynes BN. The perinatal depression treatment Cascade: baby steps toward improving outcomes. J Clin Psychiatry. (2016) 77:1189–200. doi: 10.4088/JCP.15r1017473, PMID: 27780317

[75] Pinheiro RT Souza LDM Trettim JP de Matos MB Pinheiro KAT da Cunha GK. Antenatal depression: efficacy of a pre-post therapy study and repercussions in motor development of children during the first 18 months postpartum. Study: "pregnancy care, healthy baby". J Psychiatr Res. (2022) 148:63–72. doi: 10.1016/j.jpsychires.2022.01.061Litzky, PMID: 35121270 PMC8968217

